# Effects of auditory information on self-motion perception during simultaneous presentation of visual shearing motion

**DOI:** 10.3389/fpsyg.2015.00749

**Published:** 2015-06-11

**Authors:** Shigehito Tanahashi, Kaoru Ashihara, Hiroyasu Ujike

**Affiliations:** Sensory and Perceptual Information Design Group, Human Informatics Research Institute, National Institute of Advanced Industrial Science and TechnologyTsukuba, Japan

**Keywords:** self-motion, vection, vision, audition, subjective score, random dot pattern, pink noise, perception

## Abstract

Recent studies have found that self-motion perception induced by simultaneous presentation of visual and auditory motion is facilitated when the directions of visual and auditory motion stimuli are identical. They did not, however, examine possible contributions of auditory motion information for determining direction of self-motion perception. To examine this, a visual stimulus projected on a hemisphere screen and an auditory stimulus presented through headphones were presented separately or simultaneously, depending on experimental conditions. The participant continuously indicated the direction and strength of self-motion during the 130-s experimental trial. When the visual stimulus with a horizontal shearing rotation and the auditory stimulus with a horizontal one-directional rotation were presented simultaneously, the duration and strength of self-motion perceived in the opposite direction of the auditory rotation stimulus were significantly longer and stronger than those perceived in the same direction of the auditory rotation stimulus. However, the auditory stimulus alone could not sufficiently induce self-motion perception, and if it did, its direction was not consistent within each experimental trial. We concluded that auditory motion information can determine perceived direction of self-motion during simultaneous presentation of visual and auditory motion information, at least when visual stimuli moved in opposing directions (around the yaw-axis). We speculate that the contribution of auditory information depends on the plausibility and information balance of visual and auditory information.

## Introduction

The present study focused on the effect of auditory information presented with visual information on self-motion perception. When we operate in dynamic environments, self-motion information can be obtained from the visual and auditory systems, as well as the vestibular, somatosensory, and proprioceptive systems (Lishman and Lee, 1973; Dichgans and Brandt, 1978; Warren and Wertheim, 1990; DeAngelis and Angelaki, 2012). Visual self-motion information is provided by visual global motion, or more specifically, optic flow (Gibson, 1961), and auditory self-motion information is provided by loudness changes, binaural cues, and the Doppler effect of sound (Lutfi and Wang, 1999).

Most previous studies have examined visual and auditory perception of self-motion independently. Self-motion perception induced by visual motion alone is a well-known phenomenon that has been recognized since at least the 19th century (Mach, 1875; Wood, 1895). This phenomenon was named *vection* by Fisher and Kommüler (1930) (for an overview, see Hettinger et al., 2014). Similar to visually induced self-motion, auditorily induced self-motion has long been recognized (Urbantschtsch, 1897; Dodge, 1923). For example, observers wearing eye masks perceive self-motion through auditory motion alone (Lackner, 1977; Larsson et al., 2004; Sakamoto et al., 2004; Väljamäe et al., 2004; Riecke et al., 2009; Väljamäe, 2009). Visually or auditorily induced self-motion is typically perceived in the opposite direction of visual or auditory motion, respectively.

Recently, several studies have examined the effect of visual and auditory inputs in self-motion perception by simultaneously presenting visual and auditory motion stimuli. Riecke et al. (2009), Seno et al. (2012), and Keshavarz et al. (2014) compared the magnitude of self-motion perception obtained by a visual motion stimulus alone and that obtained by simultaneous presentation of visual and auditory motion stimuli, finding that self-motion perception is facilitated when the directions of visual and auditory motion stimuli are identical. Moreover, Seno et al. (2012) reported that self-motion perception is neither inhibited nor facilitated by directional conflict between visual and auditory motion stimuli, while perceived linear direction is determined by the visual motion stimulus. These results indicate that self-motion perception can be facilitated with simultaneous presentation of visual and auditory stimuli, while visual information determines the direction of self-motion perception. However, this study did not fully examine whether directional information from auditory stimuli contributes to self-motion perception during simultaneous presentation of visual and auditory stimuli.

We investigated the possible contribution of directional information from an auditory stimulus on rotational self-motion perception during simultaneous presentation of visual and auditory rotational stimuli. To this end, we adopted visual rotational shearing motion so that visual information did not determine the direction of self-motion perception (for vertical linear shearing motion, see Kitazaki and Sato, 2003). If directional information of auditory stimuli can be used, perceived rotational direction is expected to be opposite to auditory rotation during simultaneous presentation of visual shearing motion and auditory rotation stimuli. Moreover, the visual and auditory stimuli we used were rotated around the vertical yaw axis based on previous findings (Toshima et al., 2006, 2008), which indicated that the accuracy of sound localization on the horizontal plane is greater than that on the median plane. Considering these results, greater accuracy of sound localization was expected to more easily produce self-motion perception from auditory rotation stimuli.

## Materials and methods

### Apparatus

**Figure 1:**
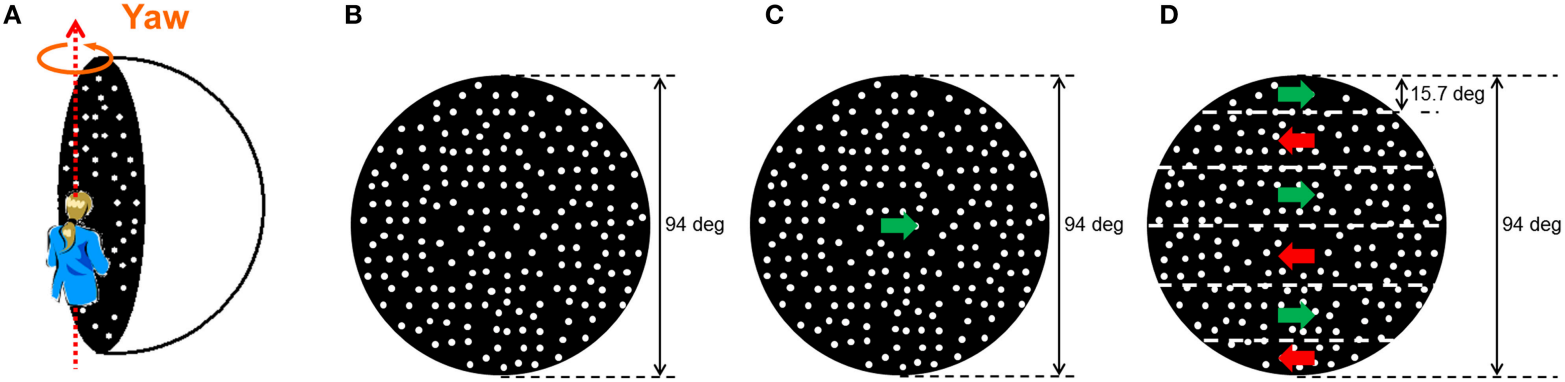
**Visual stimulus on the inside of the hemisphere screen. (A)** The random dot pattern that was rotated along the yaw axis relative to the observer was projected onto the inside of a hemisphere screen. In this experiment, the participant sat in an upright position; **(B)** stationary random dot pattern (V_SRD); **(C)** horizontal one-directional motion (V_HOM); and **(D)** horizontal shearing motion between vertically adjacent stripe-shaped areas (V_HSM).

We used an LCD projector (Epson ELP-7700) with a fisheye lens to project a visual stimulus onto the inside of a hemisphere (with a 150-cm inner diameter; Figure 1A). Headphones (BOSE TP1SB TriPort) were used to present an auditory stimulus. We installed a chair in front of the hemisphere screen surface so that an observer sitting upright had her/his feet not touching the floor. With this installation, we expected that the observer would more easily perceive self-motion by her-/himself (Lepecq et al., 1995; Wright et al., 2006; Riecke et al., 2008).

### Stimuli

#### Visual stimuli

A visual stimulus that simulated a sphere with a 150-cm diameter (Figure 1A) was rendered in real time as a computer-generated image on a Windows-based PC (Dual Core 2, 2.4 GHz) with OpenGL. The frame rate was 60 Hz, and the image size was a diameter of about 94° from a viewing distance of about 70 cm; the vantage point was the center of perspective projection. The rendered visual image was a random dot pattern consisting of white dots (diameter of dot: 1.39°; luminance: 3.03 cd/m^2^) on a black background (0.60 cd/m^2^) that was generated on the inner surface of the sphere. The white dots accounted for 33% of the area of the inner surface of the sphere. The fixation point (a red dot; diameter of dot: 2.46°; luminance: 1.05 cd/m^2^) was presented on the center of the inner surface of the sphere. The visual stimulus simulated rotation of the observer around the yaw axis. Both directions of rotation around the yaw axis were used. The rotation velocity was held constant at 36°/s. The velocity, 36°/s, was determined based on especially the auditory condition that tends to produce self-motion perception. Actually, the previous studies that examined auditorily induced self-motion perception used a stimulus velocity of 30°/s (Riecke et al., 2009) and 40°/s (Larsson et al., 2004). The specific value, 36°/s, was chosen in order to produce a stimulus between the values used in previous studies.

We used four different types of visual stimulus condition. The first was a no-visual-stimulus (V_NRD) condition, in which the observer wore an eye mask. In the other three conditions, three different moving patterns of random dots were presented: (i) stationary (V_SRD; Figure 1B), (ii) horizontal one-directional motion (V_HOM; Figure 1C), and (iii) horizontal shearing motion between vertically adjacent stripe-shaped areas (V_HSM; Figure 1D). The horizontal shearing motion was produced by six vertically adjacent striped areas, each of which used random dot motion in opposite directions relative to each other; the striped areas had a width of approximately 15.7°, and no visual border line between the adjacent areas.

#### Auditory stimuli

**Figure 2:**
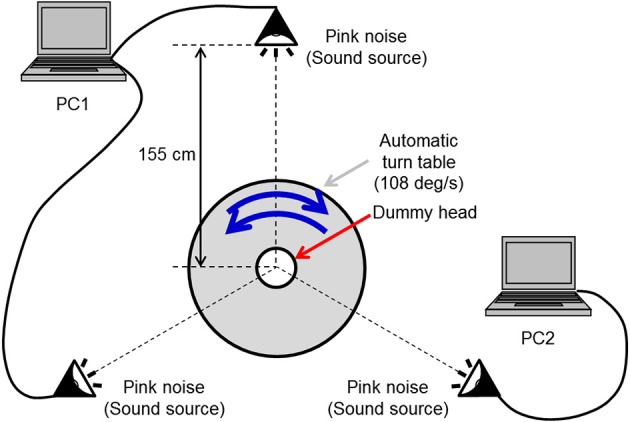
**The rotational auditory stimulus**. A pink noise was output by two notebook computers through three loudspeakers located at an equal distance and an equal angle from the rotating base. This auditory stimulus was recorded by a dummy head with a binaural microphone.

The auditory stimulus was a pink noise presented through headphones. We adopted this auditorily abstract stimulus corresponding to visual random dot stimulus. A-weighted sound pressure level for the observer was 64 dB. Both rotation directions around the yaw axis were used. The rotational auditory stimulus was produced with the following processes. First, a pink noise was synthesized and generated by two notebook computers presented from three loudspeakers (Maxer-denki MSW-SB) located at an equal distance and an equal angle from the rotating base (SHIMPO RK-5T). Second, this auditory stimulus was recorded by a dummy head with a binaural microphone (Audio-Technica AT9903; Figure 2). This dummy head was installed on the rotation center of the rotating base, and it was one-third the size of an averaged normal human head. To equalize a frequency characteristic of this dummy head to that of a normal dummy head, we converted the rotational velocity of the rotating base and the pitch of the recorded auditory stimulus. The velocity was constant at 108°/s and the pitch was one-third of the original pink noise. Then, the recorded sound was played at one-third normal speed so that the rotational velocity of the auditory stimulus became constant at 36°/s, identical to that of the visual stimulus.

We used three different types of auditory stimulus condition. The first was the no-auditory-stimulus (A_NPN) condition. In the other two conditions, two different moving patterns of the pink noise were presented: (i) stationary (A_SPN) and (ii) horizontal one-directional motion simulating rotation of the sound source around the yaw axis (A_HOM).

### Procedure

After providing informed written consent, participants were explained and experienced the self-motion perception induced by visual and auditory rotational stimulus alone. The explanation of the perception was given as an experience that participants would perceive as though their own body was rotated around an axis. Then, participants were exposed to a horizontal one-directional visual and auditory motion stimuli, such as in V_HOM + A_NPN or V_NRD + A_HOM conditions, for 1 min. After the exposure, all the participants reported that they perceived visually induced self-motion in the V_HOM + A_NPN condition. However, not all participants reported perceiving auditorily induced self-motion in the V_NRD + A_HOM condition.

Before each trial begun, participants were adapted to darkness for 10 min by sitting in a quasi-dark room while wearing an eye mask. After the adaptation, the participant wore headphones, and a 130-s experimental trial begun. At the beginning of each trial, a stationary image without an auditory stimulus was presented for 10 s, followed by a stimulus combining visual and auditory stimuli for 120 s.

The stimulus conditions were combinations of the four visual stimulus conditions and the three auditory stimulus conditions. However, we excluded three of the above combinations, V_NRD + A_NPN, V_NRD + A_SPN, and V_SRD + A_NPN, resulting in nine different experimental conditions (Table 1). The sign “+” here represents the combination of visual and auditory stimulus conditions.

**Table 1:** **Detailed trial numbers in each experimental condition**.

		**Type of visual stimulus**							
		**Non- visual-stimulus (V_NRD)**		**Stationary (V_SRD)**		**Horizontal one-directional motion (V_HOM)**		**Horizontal shearing motion (V_HSM)**	
Type of auditory stimulus
	Non- auditory-stimulus (A_NPN)		0		0	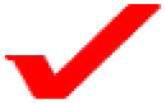	2	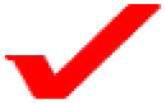	2
	Stationary (A_SPN)		0	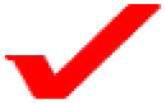	2	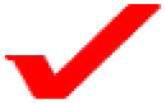	2	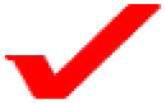	2
	Horizontal one-directional -motion (A_HOM)	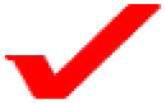	2	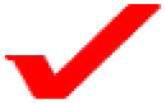	2	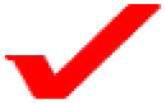	4	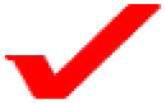	4

*The bottom-right number in each cell is the detailed trial number for each experimental condition*.

There were 22 experimental trials for each observer; these trials were performed in a randomized order across participants. The detailed trial numbers for each experimental condition are presented in Table 1. These were determined as follows: (a) two trials were assigned to V_HOM and V_HSM as well as A_HOM, corresponding to the two directional rotations of those stimulus conditions, and (b) two trials were assigned to the experimental condition V_SRD + A_SPN. Because of (a), four trials were performed in both V_HOM + A_HOM and V_HSM + A_HOM, corresponding to the combinations of two directional rotations of both visual and auditory stimulus conditions.

To measure the direction and strength of self-motion perception, we adopted a continuous subjective measurement during each trial. The continuous measurements were adopted in accordance with previous studies showing that observers often experienced intermittent self-motion perception induced by visual motion stimuli (Kleinschmidt et al., 2002; Tanahashi et al., 2012). During a trial, whenever the observers experienced self-motion perception, they continuously indicated the change in the direction and strength of self-motion perception using a subjective response box to evaluate perception on a 7-point scale (Table 2). In Table 2, “+” represents self-motion in a clockwise (CW) direction around the yaw axis, and “-” represents self-motion perceived in a counterclockwise (CCW) direction around the yaw axis. The value “0” indicates that no self-motion was perceived, and “3” indicates that the perceived self-motion could not be differentiated from real physical motion. Participants were able to clearly indicate the value of their self-motion perception without looking at the subjective response box because a gap, which was tactually perceived, was provided for each self-motion perception value on a linear potentiometer. These data were recorded at a sampling rate of 60 Hz.

**Table 2:** **Detailed 7-point scale of the change in direction and strength of self-motion perception**.

Clockwise direction around the yaw axis	+3	The perceived self-motion could not be differentiated from real physical motion
	+2	
	+1	
	0	No self-motion was perceived
Counterclockwise direction around the yaw axis	−1	
	−2	
	−3	The perceived self-motion could not be differentiated from real physical motion

Upon completion of these tasks, each participant rested for 10 min in the quasi-dark room before advancing to the next trial. All participants completed 11 trials per day across two separate days.

### Participants

Ten adults (seven women and three men; 34.4 ± 8.15 years) participated in the study after providing informed written consent, in accordance with the provisions of the Ergonomics Experiment Policy of the National Institute of Advanced Industrial Science and Technology (AIST). The observers were free to withdraw at any time during the experiment. The experimental protocol was approved in advance by the Institutional Review Board of AIST. The observers were naïve as to the purpose of the experiment, and had normal or corrected-to-normal visual acuity and normal hearing. Visual acuity was tested using the standard optotype of Landolt rings at 5 m and another optotype at 30 cm. Stereo acuity was tested using the stereo test chart (Stereo Optical Randot® Stereotest). Standard pure-tone audiometry was conducted using an audiometer (RION AA-58). Participants did not have eye or ear disease.

## Results

We examined the effect of auditory information presented with visual information on self-motion perception using the three resulting values: the strength, duration, and onset latency of self-motion perception. These values were extracted from the temporal variations in self-motion strength reported by participants. For each experimental trial, strength was averaged and duration was summed for each direction of self-motion perception. Onset latency was obtained using the time from the onset of visual and auditory stimulus presentation to the first onset of self-motion perception. When the observers did not perceive self-motion, the value of onset latency of self-motion perception was excluded from averaging and other statistical calculation. These three values were each averaged across participants.

When the visual stimulus of stationary random dot patterns and the auditory stimulus of stationary pink noise were presented simultaneously (V_SRD + A_SPN), none of the participants perceived self-motion in any of the experimental trials (zero out of 20 trials).

### When visual stimulus moved in opposing directions (shearing motion)

**Figure 3:**
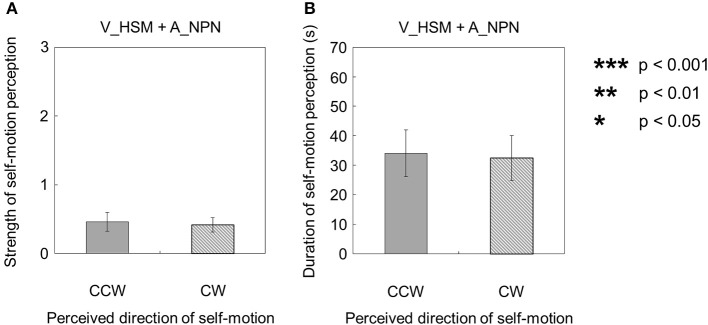
**Averaged values of the (A) strength and (B) duration of self-motion perception in the condition combining visually horizontal shearing motion (V_HSM) and auditorily horizontal one-directional motion (A_HOM)**. The values are plotted by clockwise (CW) and counterclockwise (CCW) directions. The error bars represent standard error (SE).

When the visual stimulus with a horizontal shearing motion and no auditory stimulus was presented (V_HSM + A_NPN), self-motion was perceived in both rotation directions around the yaw axis (Figures 3A,B). The strength of self-motion was not significantly different regardless of whether self-motion perception was CW or CCW [*t*_(9)_ = 0.025, *p* = 0.98, *d* = 0.011]. Moreover, the duration of self-motion episodes in each stimulus condition was not significantly different across directions of self-motion perception [*t*_(9)_ = 0.41, *p* = 0.69, *d* = 0.22].

**Figure 4:**
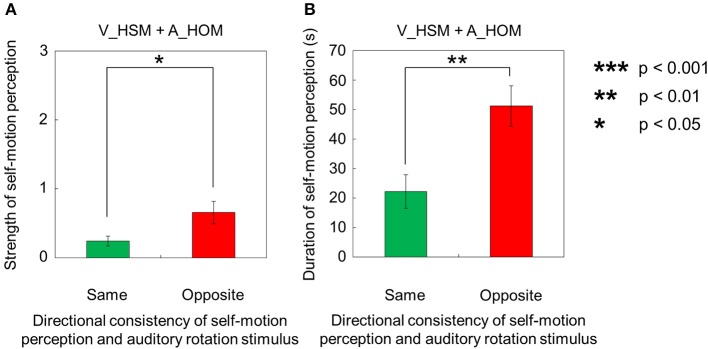
**Averaged values of the (A) strength and (B) duration of self-motion perception in the condition combining visually horizontal shearing motion (V_HSM) and no-auditory-stimulus (A_NPN)**. The values are plotted by the directional consistency of self-motion perception and the auditory rotation stimulus. The error bars represent standard error (SE).

However, when the visual stimulus with a horizontal shearing motion and the auditory stimulus with a horizontal one-directional motion were presented simultaneously (V_HSM + A_HOM), self-motion was perceived stronger and longer in the opposite direction of the auditory rotation stimulus (Figures 4A,B). The strength and duration of self-motion that was perceived in the opposite direction of the auditory rotation stimulus were significantly stronger and longer than those perceived in the same direction as the auditory rotation stimulus [strength: *t*_(9)_ = 2.38, *p* = 0.041, *d* = 0.99; duration: *t*_(9)_ = 2.83, *p* = 0.02, *d* = 1.37].

**Figure 5:**
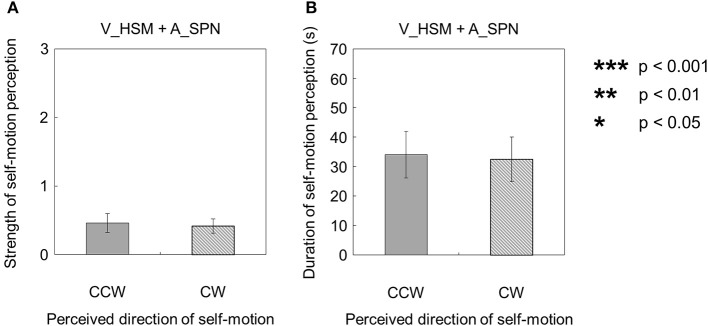
**Averaged values of the (A) strength and (B) duration of self-motion perception in the condition combining visually horizontal shearing motion (V_HSM) and auditorily stationary pink noise (A_SPN)**. The values are plotted by clockwise (CW) and counterclockwise (CCW) directions. The error bars represent standard error (SE).

Moreover, when the visual stimulus with a horizontal shearing motion and the auditory stimulus with a stationary pink noise were presented simultaneously (V_HSM + A_SPN), the strength and duration of self-motion were not significantly different, regardless of the direction of the self-motion perception [strength: *t*_(9)_ = 0.23, *p* = 0.82, *d* = 0.11; duration: *t*_(9)_ = 0.11, *p* = 0.91, *d* = 0.060; Figures 5A,B].

**Figure 6:**
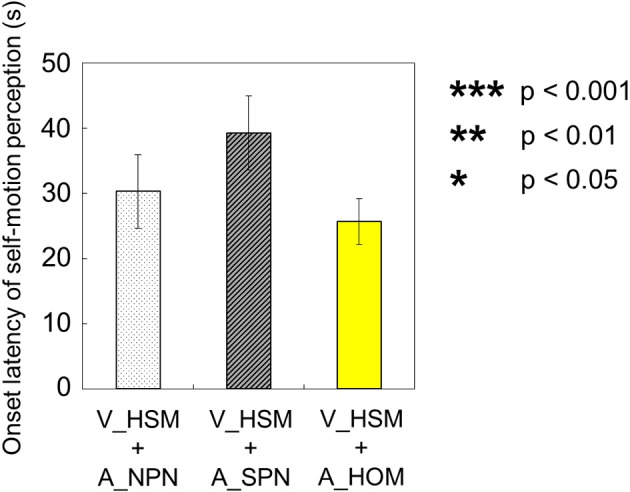
**Averaged values of the onset latency of self-motion perception in the three experimental conditions including visual shearing motion**. In each graph, the left bar shows the condition combining visually horizontal shearing motion (V_HSM) and no auditory stimulus (A_NPN). The middle bar is the combination of visual horizontal shearing motion between the vertically adjacent stripe-shaped area (V_HSM) and stationary pink noise (A_SPN). The right bar is the combination of the vertically adjacent stripe-shaped area (V_HSM) and horizontal one-directional motion simulating rotation of pink noise around the yaw axis (A_HOM). The error bars represent standard error (SE).

The onset latency of self-motion perception were compared among the above three experimental conditions (V_HSM + A_NPN vs. V_HSM + A_HOM vs. V_HSM + A_SPN). To do this, we averaged the values of onset latency across observers for each experimental condition. We found no significant differences using a One-Way repeated measures ANOVA [onset latency: *F*_(2, 27)_ = 1.78, *p* = 0.19, ${\hat{\eta }}^{2}$ = 0.12; Figures 6A–C]. All participants perceived self-motion among the above three experimental conditions.

### When either the visual or the auditory stimulus moved in one direction

**Figure 7:**
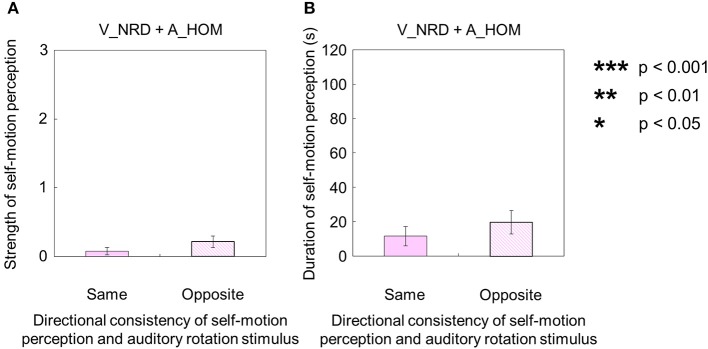
**Averaged values of the (A) strength and (B) duration of self-motion perception in the condition combining no visual stimulus (V_NRD) and auditory horizontal one-directional motion (A_HOM)**. The values are plotted the directional consistency of self-motion perception and auditory rotation stimulus. The error bars represent standard error (SE).

When either the visual or auditory stimulus was rotated horizontally in a one-directional motion without the counterpart of the stimulus (visual stimulus alone: V_HOM + A_NPN; auditory stimulus alone: V_NRD + A_HOM), participants tended to perceive self-motion. First, for the auditory stimulus with no visual stimulus (V_NRD + A_HOM), five participants perceived self-motion. The duration and strength of auditorily induced self-motion in opposite direction of the auditory rotation stimulus was significantly greater than zero [strength: *t*_(9)_ = 2.45, *p* = 0.036, *d* = 1.16; duration: *t*_(9)_ = 2.45, *p* = 0.037, *d* = 1.23]. Among them, three participants always perceived self-motion in the opposite direction of the auditory rotation stimulus; the other two did not always perceive in the opposite direction of the auditory rotation stimulus throughout the trials. The duration and strength of auditorily induced self-motion were not significantly different, regardless of the direction of self-motion perception [strength: *t*_(9)_ = 1.33, *p* = 0.22, *d* = 0.58; duration: *t*_(9)_ = 1.75, *p* = 0.11, *d* = 0.39; Figures 7A,B].

**Figure 8:**
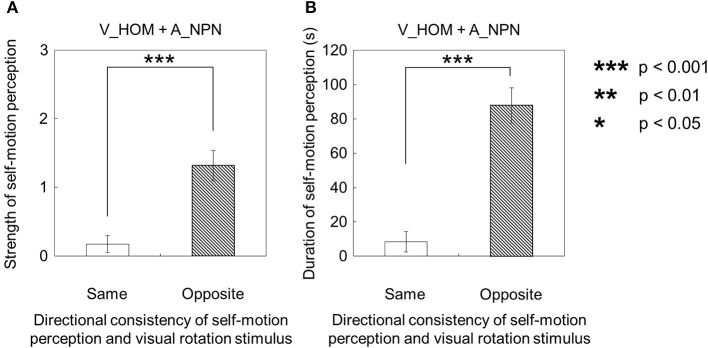
**Averaged values of (A) strength and (B) duration of self-motion perception in the condition combining visual horizontal one-directional motion (V_HOM) and no-auditory-stimulus (A_NPN) conditions**. The values are plotted by separating the directional consistency of self-motion perception and the visual rotation stimulus. The error bars represent standard error (SE).

**Figure 9:**
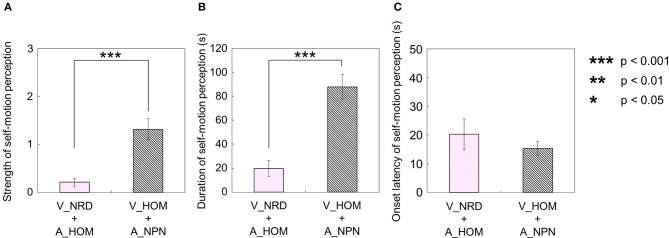
**Averaged values of (A) strength, (B) duration, and (C) onset latency of self-motion perception in two experimental conditions**. In each graph, the left bar shows the condition combining no visual stimulus (V_NRD) and auditory horizontal one-directional motion (A_HOM). The right bar shows the combination of visual horizontal one-directional motion (V_HOM) and no auditory stimulus (A_NPN). The error bars represent standard error (SE).

Second, for the visual stimulus with no auditory stimulus (V_HOM + A_NPN), all participants perceived self-motion. Among them, nine participants always perceived self-motion in the opposite direction of the auditory rotation stimulus; the other one did not always perceive in the opposite direction of the auditory rotation stimulus throughout the trials. In this experimental condition, the strength and duration of visually induced self-motion that was perceived in the opposite direction of the visual rotation stimulus were significantly longer and stronger than those perceived in the same direction as the visual rotation stimulus [strength: *t*_(9)_ = 3.45, *p* = 0.0073, *d* = 1.91; duration: *t*_(9)_ = 6.00, *p* = 0.0011, *d* = 2.82; Figures 8A,B]. Moreover, we compared the strength and duration of perceived self-motion across the above two experimental conditions. To this end, we averaged the amount of time that the participants experienced self-motion in the opposite direction of visual/auditory motion and the mean strength of perceived self-motion during the time, and averaged each of the above values across trials by either the visual or auditory information. We then found that the strength and duration of visually induced self-motion were stronger and longer than those of auditorily induced self-motion [strength: *t*_(9)_ = 4.20, *p* < 0.001, *d* = 1.98; duration: *t*_(9)_ = 4.51, *p* = 0.0015, *d* = 1.80; Figures 9A,B]. However, the onset latency of self-motion was not significantly different regardless of whether self-motion perception was induced by the visual or the auditory stimulus, which is indicated by the paired-samples *t*-tests using the data of participants who experienced self-motion perception in both conditions [^1^ *t*_(4)_ = 1.55, *p* = 0.20, *d* = 0.47; Figure 9C].

Similarly, when either the visual or auditory stimulus was rotated horizontally in a one-directional motion with the stationary stimulus of the counterpart (auditory rotation stimulus with visual stationary stimulus: V_SRD + A_HOM; visual rotation stimulus with auditory stationary stimulus: V_HOM + A_SPN), participants tended to perceive self-motion. First, for the auditory stimulus with stationary visual stimulus (V_SRD + A_HOM), six participants perceived self-motion. Among them, four participants always perceived self-motion in the opposite direction of the auditory rotation stimulus; the other two did not always perceive in the opposite direction of the auditory rotation stimulus throughout the trials. The duration and strength of auditorily induced self-motion in opposite direction of the auditory rotation stimulus was not significantly greater than zero [strength: *t*_(9)_ = 1.42, *p* = 0.19, *d* = 0.64; duration: *t*_(9)_ = 1.42, *p* = 0.19, *d* = 0.64]. The duration and strength of auditorily induced self-motion were not significantly different, regardless of the direction of self-motion perception [strength: *t*_(9)_ = 1.54, *p* = 0.16, *d* = 0.46; duration: *t*_(9)_ = 1.37, *p* = 0.20, *d* = 0.42]. Second, for the visual stimulus with stationary auditory stimulus (V_HOM + A_SPN), all participants perceived self-motion. Among them, seven participants always perceived self-motion in the opposite direction of the auditory rotation stimulus; the other three did not always perceive in the opposite direction of the auditory rotation stimulus throughout the trials. In this experimental condition, the strength and duration of visually induced self-motion that was perceived in the opposite direction of the visual rotation stimulus were significantly longer and stronger than those perceived in the same direction as the visual rotation stimulus [strength: *t*_(9)_ = 6.55, *p* < 0.001, *d* = 2.16; duration: *t*_(9)_ = 9.00, *p* < 0.001, *d* = 3.15]. Moreover, we compared the strength and duration of perceived self-motion across the above two experimental conditions. To this end, we averaged the amount of time that the participants experienced self-motion in the opposite direction of visual/auditory motion and the mean strength of perceived self-motion during the time, and averaged each of the above values across trials by either the visual or auditory information. We then found that the strength and duration of visually induced self-motion were stronger and longer than those of auditorily induced self-motion [strength: *t*_(9)_ = 7.23, *p* < 0.001, *d* = 2.31; duration: *t*_(9)_ = 11.6 *p* < 0.001, *d* = 3.47]. However, the onset latency of self-motion was not significantly different regardless of whether self-motion perception was induced by the two experimental conditions above, which is indicated by the paired samples *t*-tests using the data of participants who experienced self-motion perception in both conditions[^2^ *t*_(3)_ = 1.04, *p* = 0.37, *d* = 0.28].

**Figure 10:**
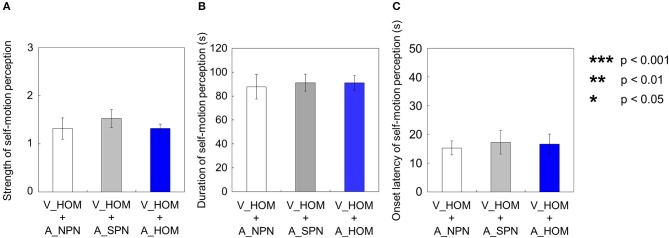
**Averaged values of the (A) strength, (B) duration, and (C) onset latency of self-motion perception in the three experimental conditions including one-directional visual rotation**. In each graph, the left bar shows the condition combining visual horizontal one-directional motion (V_HOM) and no auditory stimulus (A_NPN). The middle bar shows the combination of visual horizontal one-directional motion (V_HOM) and stationary pink noise (A_SPN). The right bar shows the combination of visual horizontal one-directional motion (V_HOM) and auditory horizontal one-directional motion (A_HOM). The error bars represent standard error (SE).

To examine the effects of existence of stationary visual stimulus on auditory-induced self-motion perception, we compared each of the three values—strength, duration, and onset latency—of self-motion perception across the two different experimental conditions (V_NRD + A_HOM vs. V_SRD + A_HOM). To do this, we averaged amount of time that the participants experienced self-motion in the opposite direction of visual/auditory motion and the mean strength of perceived self-motion during the time, and averaged each of the above values across trials by auditory information. We found no significant differences between the two experimental conditions using a paired-samples *t*-test [strength: *t*_(9)_ = 1.88, *p* = 0.092, *d* = 0.84; duration: *t*_(9)_ = 1.97, *p* = 0.081, *d* = 0.82; onset latency: ^3^ *t*_(2)_ = 0.028, *p* = 0.98, *d* = 0.0071; Figures 10A–C].

Finally, to examine the effect of existence of visual rotational shearing motion on auditory-induced self-motion perception, we compared each of the three values—strength, duration—of self-motion perception across the two different experimental conditions (V_NRD + A_HOM vs V_HSM + A_HOM). The strength and duration of self-motion perception in the V_HSM + A_HOM condition were stronger and longer than that in the V_NRD + A_HOM condition [strength: *t*_(9)_ = 2.53, *p* = 0.032, *d* = 0.75; duration: *t*_(9)_ = 2.65, *p* = 0.026, *d* = 0.84]. However, the onset latency of self-motion was not significantly different regardless of whether self-motion perception was induced by the above two experimental conditions, which is indicated by the paired samples *t*-tests using the data of participants who experienced self-motion perception in both conditions [^4^ *t*_(4)_ = 0.43, *p* = 0.69, *d* = 0.096].

### When both visual and auditory stimuli moved in one direction

**Figure 11:**
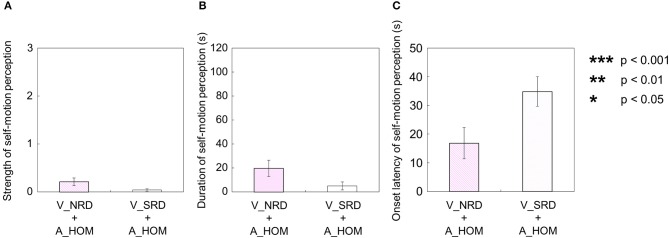
**Averaged values of the (A) strength, (B) duration, and (C) onset latency of self-motion perception in the two experimental conditions including one-directional auditory rotation**. In each graph, the left bar shows the condition combining no visual stimulus (V_NRD) and the auditory horizontal one-directional motion (A_HOM). The right bar shows the combination of the stationary random dot pattern (V_SRD) and the auditory horizontal one-directional motion (A_HOM). The error bars represent standard error (SE).

When both the visual and auditory stimuli of horizontal one-directional motion were presented (V_HOM + A_HOM), all participants predominantly perceived self-motion in the opposite direction of the visual rotation stimulus (39 out of 40 trials). The strength, duration, and onset latency of self-motion were not significantly different regardless of the consistency of rotational direction between visual and auditory stimuli [strength: *t*_(9)_ = 0.28, *p* = 0.79, *d* = 0.065; duration: *t*_(9)_ = 1.32, *p* = 0.22, *d* = 0.083; onset latency: *t*_(9)_ = 0.30, *p* = 0.77, *d* = 0.080; Figures 11A–C].

**Figure 12:**
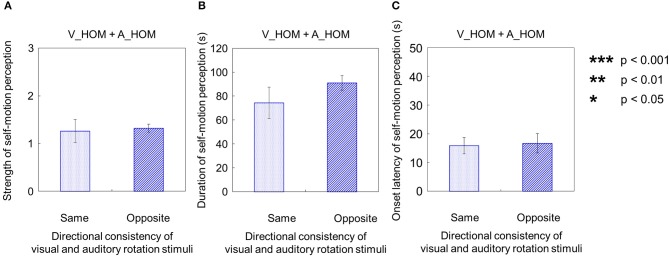
**Averaged values of the (A) strength, (B) duration, and (C) onset latency of self-motion perception in the condition combining visual horizontal one-directional motion (V_HOM) and auditory horizontal one-directional motion (A_HOM)**. The values are plotted by separating directional consistency of the visual and auditory rotation stimuli. The error bars represent standard error (SE).

We compared each of the three values—strength, duration, and onset latency—of self-motion perception across the three different experimental conditions (V_HOM + A_NPN vs. V_HOM + A_SPN vs. V_HOM + A_HOM). To do this, we averaged amount of time that the participants experienced self-motion in the opposite direction of visual/auditory motion and the mean strength of perceived self-motion during the time, and averaged each of the above values across trials by visual information. We found no significant differences in those values among the three experimental conditions using a One-Way repeated measures ANOVA [strength: *F*_(2, 27)_ = 0.41, *p* = 0.67, ${\hat{\eta }}^{2}$ = 0.029; duration: *F*_(2, 27)_ = 0.82, *p* = 0.45, ${\hat{\eta }}^{2}$ = 0.057; onset latency: *F*_(2, 27)_ = 0.85 × 10^−2^, *p* = 0.92, ${\hat{\eta }}^{2}$ = 0.0062; Figures 12A–C].

## Discussion

This study indicated that auditory motion information determined the perceived direction of self-motion during simultaneous presentation of visual and auditory motion information, at least when visual stimuli moved in opposing direction (around the yaw-axis). Moreover, our results indicated that auditory motion information can be enhanced to contribute to self-motion perception when presented with a visual motion stimulus, as the strength and duration of self-motion perception with auditory motion information alone (in the V_NRD + A_HOM condition) was weaker and shorter than those of auditory motion information presented with visual shearing motion (in the V_HSM + A_HOM condition).

Previous studies reported that auditory motion information facilitates self-motion perception when the directions of visual and auditory motion stimuli are identical (Riecke et al., 2009; Seno et al., 2012; Keshavarz et al., 2014). However, those studies also indicated that auditory motion information is weaker than visual motion information in producing self-motion perception. When the directions of visual and auditory motion information conflict with each other, visual information dominates self-motion perception. The strength and direction of perceived self-motion are the same as those when visual motion information is presented alone (Seno et al., 2012).

Our results also indicated that auditory motion information is weaker than visual information for producing self-motion perception in two respects: (1) visual information dominated self-motion perception when visual and auditory motion information were simultaneously presented in conflicting directions, and (2) self-motion perception produced by auditory motion information alone was weaker than was that produced by visual motion information alone. Item (1) was confirmed based on two different results. First, the direction of self-motion perception was determined by visual information when simultaneously presented visual and auditory motion directions conflicted with each other. Second, the strength, duration, and onset latency of self-motion perception induced by simultaneous visual and auditory yaw motion information, conflicting with each other in terms of rotational direction, were not significantly different from those obtained by visual yaw motion alone. This result was the same as that of Seno et al. (2012), who used different types of motion from those employed in the present study. Using radially expanding visual motion and weakening pure tone sounds, Seno et al. (2012) indicated that self-motion perception was neither inhibited nor enhanced when the directions of visual and auditory stimulus rotation were opposite.

Item (2) was also confirmed by two different findings. First, the strength and duration of self-motion perception produced by visual motion information alone were significantly stronger and longer than were those produced by auditory stimuli alone, while the onset latencies were not significantly different from each other. This finding would be further supported by the results of comparing between the V_HOM +A_SPN and V_SRD + A_HOM conditions. Second, self-motion perception induced by auditory motion information alone was not strong enough to persist. Five of the 10 participants perceived self-motion with auditory motion information alone. This ratio is consistent with previous reports, which range from 20 to 60%, depending on the auditory motion stimulus used (Lackner, 1977; Larsson et al., 2004). Moreover, auditory one-directional motion stimuli did not consistently induce self-motion opposite to the direction simulated by the stimulus. This is consistent with Sakamoto et al. (2004), who used auditorily linear motion stimuli. This finding might be explained by participants incorrectly perceiving the rotational direction simulated by the auditory stimulus. In fact, the literature suggests that perceived rotational direction of auditory stimuli does not necessarily coincide with that of the actual stimulus, probably because of front-back confusion (Young, 1931; Wallach, 1939, 1940). Although we confirmed that participants perceived the direction correctly before the experimental trials began, we cannot be certain of the perceived direction of the auditory stimulus itself.

Our results did not indicate that auditory motion information facilitates self-motion perception when the directions of visual and auditory motion are identical, which is inconsistent with previous reports (Riecke et al., 2009; Seno et al., 2012). This difference might be attributable to differences in the plausibility and information balance of visual and auditory stimuli. First, Riecke et al. (2009) used visual stimuli of actual recorded scenes, including a fountain located at a marketplace and a corresponding auditory stimulus. This kind of auditory stimulus can easily induce self-motion perception, as the sound sources typically associated with stationary objects are more effective in triggering auditory self-motion perception than are sounds stemming from moving objects or artificial sounds like pink noise (Larsson et al., 2004; Riecke et al., 2005). Second, the predominance of visual motion information over auditory motion information can vary by the size of the visual field of view (FOV). Riecke et al. (2009) manipulated the size of the visual FOV presented simultaneously with auditory information, and found that auditory information more effectively facilitated self-motion perception with medium-sized FOV (20 × 15°) than with a large-sized FOV (54 × 45°). In contrast to Riecke et al. (2009), we used an artificial sound (i.e., pink noise) as an auditory stimulus and a visual stimulus with a diameter of about 94°, which was larger than the size used by Riecke et al. (2009). This might explain why auditory motion information was not as effective in facilitating self-motion perception during simultaneous presentation of visual and auditory information.

If the plausibility and information balance of visual and auditory stimuli varied the extent of auditory facilitation of self-motion, these factors can also vary the predominance of visual over auditory information for the perceived direction of self-motion. In our experimental conditions, auditory motion information was weaker than visual information. It was in the condition in which visual information did not specify self-motion direction that auditory motion information could determine the perceived direction of self-motion. If we could appropriately manipulate the predominance of visual over auditory motion information, auditory motion information might contribute more to determining the perceived direction of self-motion during simultaneous presentation of visual and auditory motion information.

**Figure 13:**
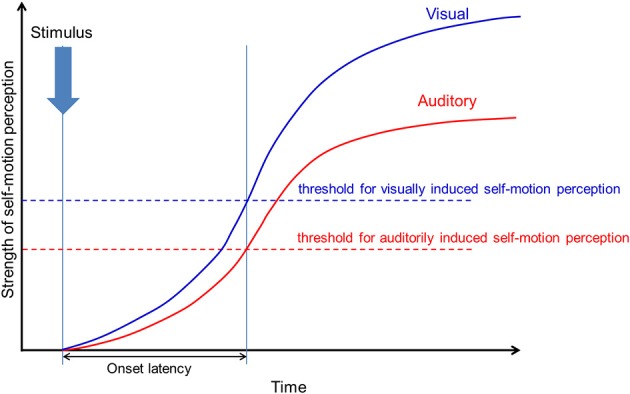
**The thresholds of visually and auditorily induced self-motion perception**. The blue line shows the strength threshold of visually induced self-motion perception. The red line shows the strength threshold of auditorily induced self-motion perception.

Our results showed no significant difference in the onset latency of visually and auditorily induced self-motion perception, while the perceived strength and duration were significantly different. This discrepancy may be explained by the difference between the strength threshold of visually and auditorily induced self-motion perception, as discussed in Tanahashi et al.'s (2007) simple model of visually induced self-motion perception. Although the strength of auditorily induced perception is weaker than that of visually induced perception, the threshold of auditorily induced perception may be lower than that of visually induced perception, allowing for the onset latency to be equal (Figure 13). This also indicates that weaker self-motion strength can be perceived with auditory information in comparison to visual information, which may be worth examining. At the same time, however, we have to consider that the number of participants in our study was rather small (*N* = 10) and test power was consequently weak (1-β = 0.42) to detect a relevant effect size (Cohen's *d* = 0.5). Thus, the non-significant results found in our study need to be treated very carefully.

We conclude that auditory motion information can determine perceived direction of self-motion during simultaneous presentation of visual and auditory motion information, at least when visual stimuli moved in opposing direction (around the yaw-axis). Moreover, considering previous studies (Riecke et al., 2009; Seno et al., 2012), we indicate that auditory information can contribute to self-motion perception when the directions of visual and auditory motion stimuli are identical. The contribution of auditory information seems to depend on the plausibility and information balance of visual and auditory information.

### Conflict of interest statement

The authors declare that the research was conducted in the absence of any commercial or financial relationships that could be construed as a potential conflict of interest.

## Footnotes

^1^When we compared the onset latency of perceived self-motion across the V_NRD + A_HOM and V_HOM and A_NPN conditions using unpaired-samples *t*-tests, the onset latency of self-motion was not significantly different regardless of whether self-motion perception was induced by the visual or the auditory stimulus [*t*_(13)_ = 0.82, *p* = 0.43, *d* = 0.48].

^2^When we compared the onset latency of perceived self-motion across the V_SRD + A_HOM and V_HOM and A_SPN conditions using unpaired-samples *t*-tests, the onset latency of self-motion was not significantly different regardless of whether self-motion perception was induced by the above two experimental conditions [*t*(12) = 2.08, *p* = 0.060, *d* = 1.33].

^3^When we compared the onset latency of perceived self-motion across the V_NRD + A_HOM and V_SRD and A_HOM conditions using unpaired-samples *t*-tests, the onset latency of self-motion was not significantly different regardless of whether self-motion perception was induced by the above two experimental conditions [*t*(7) = 1.50, *p* = 0.18, *d* = 1.13].

^4^When we compared the onset latency of perceived self-motion across the V_NRD + A_HOM and V_HSM and A_HOM conditions using unpaired-samples *t*-tests, the onset latency of self-motion was not significantly different regardless of whether self-motion perception was induced by the above two experimental conditions [*t*_(13)_ = 0.74, *p* = 0.47, *d* = 0.44].
